# Microplastics and Nanoplastics as Potential Metabolic Disruptors: Implications for Insulin Resistance and Type 2 Diabetes

**DOI:** 10.3390/toxics14070634

**Published:** 2026-07-21

**Authors:** Umberto Cornelli, Claudio Casella

**Affiliations:** 1 Department of Molecular Pharmacology and Therapeutics, School of Medicine, Loyola University, 2160 1st Ave, Maywood, IL 60660, USA; 2 Department of Chemistry, University of Pavia, Viale Taramelli 12, 27100 Pavia, Italy; claudio.casella01@universitadipavia.it

**Keywords:** microplastics, insulin resistance, inflammation, glucagon, microplastic drainage

## Abstract

Human biological matrices such as blood, placenta, lung tissue, and stool have been demonstrated to contain microplastics (MPs) and nanoplastics (NPs), indicating systemic dispersion and long-term environmental exposure. According to novel experimental findings, these xenobiotics may interact with pathways that overlap with the early pathophysiology of insulin resistance and metabolic syndrome, potentially serving as metabolic disruptors. High levels of MP/NP exposure are thought to alter intestinal permeability structurally, which may have an impact on enteroendocrine L-cell environments and the ensuing incretin responses. In animal studies, downstream effects include altered bile acid balance and microbiome remodelling, which is defined by a decrease in taxa that produce short-chain fatty acids (SCFAs). These xenobiotics’ portal translocation provides a plausible mechanism for subclinical hepatic inflammation, which may function in tandem with conventional risk factors to disrupt normal metabolic signalling. We consider the translational theory of “MP drainage” as a conceptual approach to lower intestinal particle bioavailability in order to address these theoretical interactions. Nevertheless, its long-term safety, metabolic advantages, and therapeutic effectiveness are yet unknown and require further confirmation. This perspective provides a framework for creating hypotheses that will direct future experimental and epidemiological studies in environmental metabolic toxicity.

## 1. Introduction

Water, food, air, and biological tissues have all been demonstrated to contain microplastics (MPs), which have become ubiquitous in the human environment [1]. Their capacity to gather in organs, interact with immunological and metabolic systems, and penetrate across epithelial barriers has generated concerns about their potential function in chronic illnesses that are not communicable. Due to their connection to endocrine disruption and persistent low-grade inflammation, type 2 diabetes (T2D) and its precursor, metabolic syndrome, constitute extremely important endpoints among these disorders. Nevertheless, since MPs and nanoplastics (NPs) do not constitute a consistent exposure category, assessing the toxicological risk of these xenobiotics is extremely difficult. Downstream biological effects are significantly modulated by particle heterogeneity, which includes polymer type, size distributions, geometric form, surface chemistry, environmental ageing, and structural additions [2,3]. It is also important to recognise that the inert core polymer could not be the only cause of the observed metabolic disruptions. Co-transported endocrine-disrupting chemicals (EDCs), adsorbed persistent organic pollutants (POPs), and plastic additives (such as phthalates and bisphenols) are all extremely bioavailable and may be far more biologically active than the plastic particles themselves. By directly attaching to nuclear receptors and exacerbating metabolic toxicity, these chemical vectors function through a “Trojan horse” effect [3,4,5]. Importantly, obesity, physical inactivity, ageing, genetics, and eating habits are the main causes of T2D, which is one of the most complex medical conditions. It is still completely unknown how significantly the risk of T2D might be in relation to exposure to MPs/NPs in comparison to known risk factors like obesity, excessive calorie intake, or physical inactivity, and there is a lack of direct human epidemiological evidence.

In actuality, overlapping genetic predispositions, ageing, and fundamental lifestyle variables, including chronic excess and physical inactivity, are associated with T2D, a classic multifactorial metabolic disease [6]. Due to the lack of long-term human epidemiological cohorts, it is presently impossible to quantify the precise attributable risk fraction of environmental plastic particles within this complicated aetiology [7]. MPs/NPs must therefore be rigorously understood as environmental metabolic disruptors that may function as a secondary “hit”—or metabolic accelerant—operating in conjunction with pre-existing pathophysiological vulnerabilities, rather than establishing direct aetiology [8]. According to this terminology, systemic meta-flammation is a low-grade, persistent inflammatory state that the innate immune system orchestrates in reaction to environmental or metabolic stress.

Hepatic insulin resistance, excess glucagon, incretin dysfunction, and systemic metaflammation are certain of the established mechanisms contributing to early T2D pathogenesis that overlap with these processes [9,10,11].

It differs from classical acute inflammation in that it is not associated with an established infectious aetiology. We also propose a conceptual technique of “MP drainage” to reduce intestinal MP concentration and perhaps attenuate downstream metabolic disturbances. This perspective employs novel experimental data to provide a testable model for metabolic toxicity in the future, as opposed to synthesising existing clinical facts.

## 2. Intestinal Barrier Dysfunction and Impaired Incretin Signalling

MPs penetrate the body predominantly through the gastrointestinal system. MPs induce intestinal permeability, tight junction disruption, and epithelial stress, according to previous studies [12,13]. These alterations facilitate the paracellular translocation of particles and associated contaminants to circulate freely, leading to local inflammation and compromising the function of enteroendocrine L cells. GLP-1 release from L cells is essential for postprandial glucose control. By affecting food sensing, compromising epithelial integrity, and altering the luminal environment, MPs inhibit the release of GLP-1 [14,15,16]. Early dysglycemia, reduced insulin production, and increased postprandial glycaemic excursions all result from reduced GLP-1. Additionally, bile acid signalling—a key regulator of GLP-1 synthesis via FXR and TGR5 receptors—is disrupted by MPs. Models exposed to MPs demonstrated altered bile acid composition and receptor activation [17,18,19], and bile acid dysfunction is considered to decrease incretin responsiveness [20,21,22]. Overall, experimental data suggest that exposure to plastic fragments represents a plausible mechanistic pathway that may contribute to an early disruption of the incretin axis. All of these findings indicate that MPs/NPs modify the intestinal barrier structurally and affect bile acid-mediated L-cell signalling through MP/NP exposure. This early dysregulation provides a physiologically plausible paradigm through a process in which exposure to MPs/NPs in the environment may potentially lead to metabolic disorders, a connection requiring conclusive clinical evidence.

## 3. Microbiome Remodelling and Metabolic Signalling

MPs affect microbial metabolic pathways and inhibit species that generate short-chain fatty acids (SCFAs) in the gastrointestinal (GI) tract microbiome [23]. SCFAs, specifically butyrate, are critical for promoting GLP-1 production, controlling inflammation, and preserving epithelial integrity [24,25,26,27]. Decreased availability of SCFA correlates to increased intestinal permeability, decreased L cell responsiveness, reduced insulin sensitivity and systemic inflammation.

Additionally, MPs interact with the metabolism of bile acids, altering the balance of primary to secondary bile acids and influencing FXR/TGR5 signalling [21]. These pathways are essential for energy expenditure, lipid metabolism, and glucose homeostasis. Consequently, microbiota alteration is hypothesised to be a contributing component within the integrated model of particle-induced metabolic disruption.

## 4. Hepatic Inflammation, Excess Glucagon, and Insulin Resistance

The portal circulation permits translocated microplastics and corresponding contaminants to pass through the liver. MPs are internalised by Kupffer cells, which induce inflammatory cascades that include oxidative stress, TNF-α, and IL-6 [28,29,30,31]. Hepatic insulin resistance is promoted, gluconeogenesis is increased, and insulin signalling is disrupted by chronic hepatic inflammation. Additionally, inflammation amplifies fasting hyperglycaemia by increasing hepatic glucagon sensitivity and stimulating glucagon secretion [32,33]. Early T2D is characterised by this glucagon-driven metabolic alteration. According to recent studies [34,35], T2D has been identified as one of the disorders correlated with MP exposure, thereby underscoring the necessity to rigorously evaluate the full spectrum of clinical pathologies potentially aggravated by environmental particle burdens [36].

## 5. Theoretical Model of Intravascular Kinetics and Nanoplastic Differentiation

Emerging experimental models indicate that MPs/NPs that enter the systemic circulation have complex kinetic characteristics that are affected by their interactions with endogenous blood carriers. MPs/NPs are hypothesised to travel across the GI tract and quickly bind to erythrocytes. In environmental toxicology, the kinetic behaviour of circulating plastic particles is still a complicated topic. While we specifically point toward the fact that there exists presently little direct human clinical data for these particular pathways, we provide an integrated conceptual model of intravascular particle dynamics to steer future empirical validation.

MPs and NPs must be distinguished clearly from each other in terms of toxicology. Due to their sub-micron size, NPs are far more capable than larger MPs of passing through biological barriers, translocating passively across membranes, entering cells, and interacting directly with intracellular organelles. As a result, NPs rather than MPs are the main drivers of the molecular interactions described inside circulation [2,3].

NPs are significantly more effective than larger MPs in reaching organelles due to their unique interfacial kinetics, enabling them to pass through cellular membranes via receptor-mediated endocytosis or passive lipid bilayer penetration [37]. Therefore, the intricate intracellular cascades, protein corona formations, and intravascular transport pathways described below should be interpreted as phenomena primarily driven by the nanoscale fraction [38], even though MPs primarily cause localised mechanical abrasion and luminal epithelial stress within the GI tract.

Within this theoretical framework, translocated NPs are hypothesised to exhibit complex interactions with blood-borne carriers. Upon entering the systemic circulation across the GI tract, NPs may associate with cellular membranes, including those of erythrocytes. Erythrocytes continuously release extracellular vesicles (EVs). It is hypothesised that NP exposure may alter standard EV biogenesis and cargo transport, serving as a conceptual mechanism for vascular endothelial signalling alterations [39].

Concurrently, it is expected that NPs may interact with circulating lipoproteins, including high-density lipoproteins (HDL), due to their extremely hydrophobic surfaces, perhaps creating a dynamic biomolecular corona (biocorona). HDL facilitates peripheral glucose metabolic signalling and controls lipid transport under normal physiological settings. This biocorona is thought to be structurally altered when NPs attach to HDL, providing a theoretical model for impaired lipoprotein functioning and receptor recognition [2,3].

This intravascular reservoir and the ensuing metabolic signalling interference remain a theoretical framework rather than a proven physiological mechanism until they are verified by thorough in vivo pharmacokinetic monitoring models.

Exposure to MPs has been demonstrated to trigger vascular endothelial dysfunction and macrophage activation through aberrant EV signalling cascades [40], whereas internalisation of NPs encourages a pro-inflammatory shift in circulating EVs that impairs recipient tissues’ peripheral insulin sensitivity [41]. Due to their altered ability to transport lipids, proteins, and regulatory nucleic acids, these MP/NP-EV complexes may interfere with intercellular signalling and metabolic balance.

Simultaneously, MPs/NPs interact with lipoproteins, especially HDL, which are continuously recycled metabolically and synthesised in the liver and GI tract. According to recent studies, MPs/NPs may affect the distribution and content of EV cargo, impacting metabolic and endocrine control pathways [42]. The binding of MPs/NPs to HDL particles is a possible method by which MPs/NPs disrupt lipid and glucose homeostasis, considering that HDL participates in reverse cholesterol transportation and regulates glucose metabolism through hepatic and peripheral signalling. The concept of a permanent intravascular reservoir with delayed clearance and ongoing recirculation is supported by the confirmation of MPs/NPs in circulating blood by human biomonitoring [1].

The systemic pro-inflammatory state can be exacerbated when MPs bind to erythrocyte-derived EVs because they can cause changes in membrane fluidity and encourage aberrant activation of vascular macrophages. Conversely, binding to HDL reduces their capacity to favourably control pancreatic beta-cell-mediated insulin production and minimises their beneficial role in reverse cholesterol transportation. This contributes to a chronic kinetic toxicity that accelerates the glycaemic instability characteristics of T2D [37].

The combined information suggests that MPs/NPs undergo recurrent transit via HDL-mediated transport and EV turnover, consequently impacting hepatic lipid binding, inflammatory tone, and insulin signalling, despite the lack of a formal physiology-based pharmacokinetics (PBPK) model. This increased kinetic burden may worsen dyslipidaemia, endothelial dysfunction, and glycaemic instability in patients with T2D, which is already defined by altered EV profiles, chronic inflammation, and reduced lipid fluxes. Consequently, erythrocyte binding, EV trafficking, and HDL recycling influence the kinetic behaviour of circulating MPs/NPs, which is a physiologically plausible factor in the metabolic deterioration of T2D.

## 6. Toxicokinetics, Biodistribution, and Cellular Interactions of NPs: The Modulating Role of Biocorona and Surface Charge

NPs pose a serious concern to human health, considering that they may be absorbed via biological barriers, disperse into the systemic circulation, and bioaccumulate inside certain organs and tissues, according to recent toxicological findings. Particle size has a negative correlation with the effectiveness of NP internalisation into target organs, including the brain, lungs, and gastrointestinal tract. Interestingly, NPs smaller than 10 nm have physical characteristics similar to those of gaseous molecules, allowing for profound tissue penetration and subsequent intracellular transfer [2]. The ability of human cells to internalise these xenobiotics has been verified by in vitro models. In particular, intestinal organoids, macrophages, and pulmonary and intestinal epithelial cells all collect polystyrene nanoplastics (PS-NPs) that range in size from 50 to 500 nm. Additionally, human physiological matrices such as whole blood, placental tissue and hepatic parenchyma have been used to quantify the systemic presence of MPs/NPs [2].

The creation of a biomolecular corona (biocorona) and the pristine surface charge are two interconnected physicochemical characteristics that largely control the toxicokinetics, biodistribution, and cytocompatibility of NPs. When NPs enter biological fluids, their high surface-area-to-volume ratio, unique surface charges, and polymeric composition enable them to interact with endogenous lipids, proteins, and nucleic acids immediately, drastically changing their original physicochemical identity. Extracellular polymeric macromolecules produced by the host’s metabolism or intracellular elements discharged into the environment constitute this corona [2,43]. Thermodynamic forces that generate two distinct layers according to binding affinity govern the adsorption of proteins onto the NP surface [2,43]. The inner layer, referred to as the hard corona, gives the NP complex structural stability through high-affinity contacts and minimal protein desorptive turnover. The soft corona, conversely, is formed by weaker, extremely dynamic protein–protein interactions that interchange quickly and form the outer layer in direct contact with the biological media [2,43,44]. Receptor-mediated cell recognition and cellular signalling pathways are two important molecular communication processes that may be disrupted by this macromolecular adsorption, which frequently causes conformational changes in the constituent proteins. As a result, the biocorona gives the NPs a new biological identity by influencing their organ accumulation, systemic clearance, bio-interface, and cellular internalisation [45]. Additionally, biocorona dynamic development may significantly alter toxicodynamic profiles, neutralising the inherent toxicity of the core polymer or producing antagonistic or synergistic toxicological effects [44].

NPs’ initial cellular interaction and subsequent pathogenicity are determined by their pristine surface charge in addition to corona dynamics. When compared to their anionic counterparts, positively charged (cationic) NPs often exhibit increased cytotoxicity [45]. Cationic NPs exhibit a strong affinity for the negatively charged phospholipids present in cell membranes due to electrostatic attraction. Particularly in non-phagocytic lineages, this interaction increases passive intracellular translocation and modifies membrane surface tension [46]. Importantly, positively charged NPs are significantly more inclined to induce intracellular oxidative stress by producing excessive amounts of reactive oxygen species (ROS), which speeds up subsequent necrotic or apoptotic processes.

## 7. Mechanisms of Cellular Internalisation and Intracellular Trafficking

NPs are mostly internalised by cells by endocytosis, passive diffusion, and direct membrane transfer [2,43,44]. Particle size ranges, usually between 40 and 200 nm, play a major role in endocytic pathways, which are active, energy-dependent activities divided into phagocytosis and pinocytosis. Phagocytosis is a specialised process that is mostly limited to specialised phagocytes such as neutrophils and macrophages. It is controlled by varied cellular uptake kinetics. Conversely, pinocytic pathways are common in all non-phagocytic lineages. These include caveolae-mediated endocytosis, which is frequently associated with macropinocytosis and internalises NPs distributed throughout the extracellular fluid, and clathrin-mediated endocytosis, which requires the formation of organised vesicles [2,43,44]. NPs may penetrate the lipid bilayer by passive diffusion, which is fuelled by direct biochemical interactions between the adsorbed biocorona and the host cell membrane, in addition to active endocytic processes. The surface properties of the core polymer, such as membrane bending elasticity, tensile modulus, and macromolecular saturation kinetics, greatly influence the hydrophobicity and interfacial behaviour provided by the biocorona [2,43,44].

NPs are first organised into compartments within vesicular structures known as early endosomes upon successful internalisation. The intracellular NP load is characterised by a variety of toxicokinetic processes, including exocytosis, direct release from early endosomes into the cytosol, and passive efflux mechanisms [47]. Nevertheless, the NP load transfers into late endosomes when early endosomes accumulate in the cytoplasm. These endosomes then combine with autophagosomes and eventually develop into lysosomes to produce autolysosomes [47]. The autophagic activity is disrupted by the continuous accumulation of NPs due to their great resistance to lysosomal hydrolase degradation. Lytic enzymes migrate into the cytoplasm as a result of this obstruction, which causes lysosomal membrane permeabilisation (LMP) [47]. The pristine or modified protein coronas of the NPs can function as seeds once they are released into the cytosolic cytoplasm, encouraging the misfolding and aggregation of endogenous cytosolic proteins. This increases intracellular damage and sets off cascades of localised cytotoxicity [2,43,44,45,46,47].

## 8. Systemic Meta-Inflammation and Peripheral Insulin Resistance

**Figure 1:**
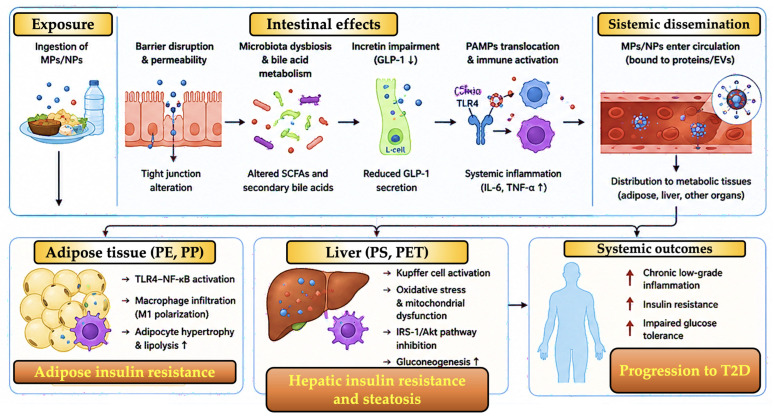
Pathophysiological mechanisms of MP/NP-induced metabolic dysfunction, leading from intestinal barrier disruption to T2D.

By increasing intestinal permeability, activating the immune system, and inducing oxidative stress, MPs promote systemic inflammation [48]. The systemic transfer of MPs/NPs and luminal pathogen-associated molecular patterns (PAMPs), particularly lipopolysaccharides (LPS) from Gram-negative bacteria, is facilitated by this increased paracellular permeability. Metabolic endotoxemia, a recognised symptom of persistent, low-grade systemic inflammation (meta-inflammation) and the ensuing insulin resistance, is directly mimicked by this mechanism [49,50] (Figure 1).

The majority of these hypothesised pathways, nevertheless, rely on multi-linked inference chains (i.e., MP-induced gut dysbiosis or localised oxidative stress isolation) rather than direct, real-world observations connecting environmental-level exposure to overt insulin resistance and clinical diabetes in vivo [51]. The following is a crucial toxicological warning. Although these experimental models provide significant biological plausibility for metabolic disturbance, it is also crucial for the field to confirm whether these interrelated molecular assumptions result in reliable clinical results under actual environmental conditions [52].

Translocated MPs and co-transported endotoxins activate the innate immune system once they enter the systemic circulation. The main mechanism of this activation is the binding of LPS to Toll-like receptor 4 (TLR4) on tissue-resident macrophages and circulating monocytes. Tumour necrosis factor-alpha (TNF-α), interleukin-1 beta, and interleukin-6 (IL-6) are among the pro-inflammatory cytokines that are secreted continuously as a result of a strong transcriptional programme orchestrated by the downstream activation of the nuclear factor kappa B (NF-κB) signalling pathway [49,50].

These mechanisms are comparable to metabolic endotoxemia, which is a recognised cause of insulin resistance [9,49,50]. Insulin signalling in muscle and adipose tissue is further hampered by oxidative stress and mitochondrial dysfunction [53,54]. Thus, prolonged exposure to MPs may be associated with early insulin resistance, decreased glucose absorption, and metabolic elasticity. The metabolic phenotype typical of early T2D is generated by a combination of these systemic effects with the hepatic and incretin pathways.

Both the systemic cytokine cascade and localised oxidative stress significantly reduce insulin signalling in peripheral target tissues, such as skeletal muscle and white adipose tissue. The phosphoinositide 3-kinase (PI3K)/protein kinase B (Akt) cascade is activated when insulin binds to its receptor under physiological conditions, activating the autophosphorylation of insulin receptor substrates (mainly IRS-1) on particular tyrosine residues. This ultimately causes the translocation of glucose transporter 4 (GLUT4) to the plasma membrane. Nevertheless, counter-regulatory intracellular stress kinases, namely c-Jun N-terminal kinase (JNK) and inhibitor of kappa B kinase beta, are activated by MP-induced systemic TNF-α and localised ROS overproduction. By mediating the heterologous serine phosphorylation of IRS-1 (such as Ser307 on human IRS-1), these activated kinases successfully dissociate the insulin receptor from subsequent PI3K/Akt activation. As a result, peripheral glucose absorption is significantly reduced, and metabolic flexibility—the ability of cells to adjust substrate oxidation to fuel availability—is reduced due to decreased GLUT4 translocation [53,54].

Importantly, the lack of long-term prospective epidemiological cohorts renders it challenging to establish a convincing causal relationship between ambient MNP exposure and T2D pathogenesis in human environmental health risk assessments. MNP exposure interacts with conventional lifestyle-driven metabolic risk factors, such as sedentary behaviour and high-fat, hypercaloric meals, rather than occurring in a physiological vacuum. MP/NP bioaccumulation is thought to function as a secondary metabolic “hit”. While physical inactivity reduces mitochondrial biogenesis and a Western diet drives initial low-grade adipocyte hypertrophy, the concurrent systemic translocation of chemically weathered MPs/NPs accelerates these pathways by triggering macrophage TLR4 activation and lysosomal membrane permeabilisation. MPs/NPs should thus be considered environmental accelerants which operate in concert with pre-existing metabolic vulnerabilities to reduce the threshold for overt peripheral insulin resistance rather than as distinct aetiologies.

## 9. MP Drainage: A Translational Intervention Hypothesis

Reducing intestinal MP concentration, or “MP drainage,” may be a beneficial approach to mitigate a metabolic disturbance given the gastrointestinal origin of MP exposure. A fascinating translational theory that aims to minimise downstream metabolic stress is the reduction of intestinal plastic particle availability by luminal sequestration, also referred to as “MP drainage”.

The MP drainage concept should currently be considered a hypothesis-generating translational strategy rather than a validated therapeutic intervention [48]. Given that current preliminary observations are largely grounded in exploratory models and pilot data generated by our research group [55,56], clinical efficacy remains unproven in robust, large-scale human populations [57]. While agents capable of binding or sequestering particles within the chyme provide a logical framework to theoretically reduce systemic inflammatory tone [57], rigorous double-blind randomised controlled trials (RCTs) are mandatory to evaluate long-term safety profiles, potential micronutrient co-adsorption risks, and definitive metabolic relevance [55,56].

**Figure 2:**
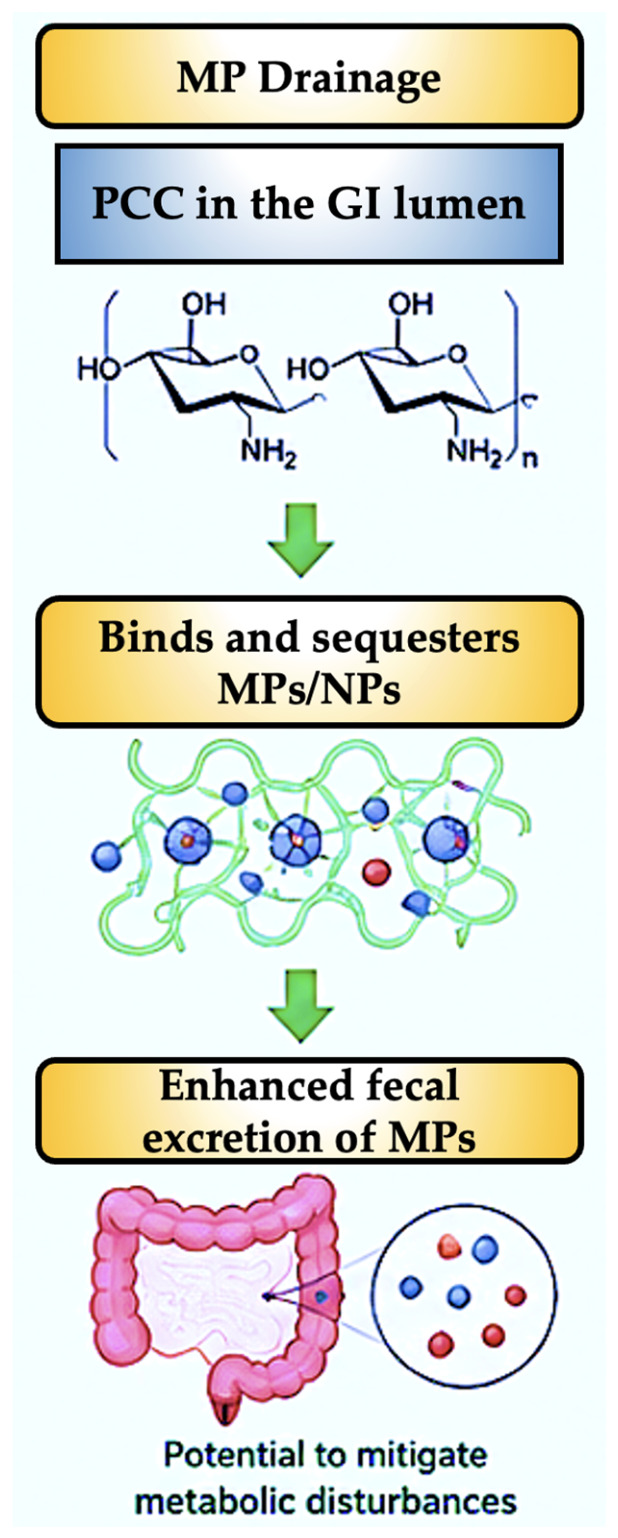
Schematic mechanism of chitosan-mediated sequestration and faecal excretion of MPs in the GI.

Early exploratory studies, such as our group’s use of a chitosan-based formulation (chitosan from Procambarus clarkii, PCC) to improve faecal excretion of particles in pilot settings, comprise the majority of the preliminary observations that are now readily available (Figure 2).

Although these preliminary trials provide a logical foundation for additional studies, people should not consider this approach to be a proven therapeutic treatment [58]. Following short-term supplementation, complementary findings from a recent study indicate a reduction in bloodstream MP levels [59]. These first findings provide credibility to the broader concept of MP drainage and call for controlled studies to establish its metabolic significance.

Theoretically, agents that bind or sequester MPs during digestion will reduce systemic inflammatory tone, limit epithelium stress, and perhaps restore GLP-1 responsiveness. Nevertheless, except when these metabolic advantages are confirmed in human interventional studies, they remain just theoretical.

It is necessary to identify specific dose schemes and possible drugs in order to advance this approach from a conceptual notion to a clinically feasible protocol. A therapeutic dosage of 1.2 g/day of high-deacetylation-degree PCC, administered orally in separate doses (i.e., 400 mg 15 min before main meals), is advised to enable adequate luminal mixing with chyme, according to preliminary human pilot findings [58].

Future translational pipelines should investigate non-absorbable oral chelators other than PCC, such as highly porous biomimetic silica particles or functionalised carbonaceous matrices that are optimised to selectively trap hydrophobic polymers (such as PE and PP) via strong hydrophobic interactions without adsorbing vital micronutrients.

The polycationic characteristics of the molecule, which might create persistent electrostatic interactions with negatively charged functional groups on the surface of MPs modified by the stomach environment, provide the basis for the mechanism of action underlying MP drainage by PCC. By encouraging the creation of non-absorbable macroaggregates trapped in the polymer matrix, this bond reduces their time in the intestinal lumen and increases their faecal evacuation. Future studies in the field of metabolic toxicology should concentrate on long-term randomised clinical trials that monitor not only the decrease in circulating MP concentration but also the normalisation of peripheral insulin resistance parameters and the restoration of enteroendocrine L-cell function markers (such as GLP-1 kinetic levels) [60].

## 10. Insulin Resistance and MPs/NPs

Adipose tissue and the liver represent two major and interconnected nodes of metabolic dysfunction in T2D, where insulin resistance (IR) develops through tissue-specific processes. Hypertrophy, hypoxia, and adipokine imbalance in adipose tissue enhance lipolysis and impair insulin signalling by promoting chronic low-grade inflammation and macrophage M1 polarisation [58]. Increased hepatic lipid infiltration, steatosis, activation of the NF-κB and SOCS pathways, and decreased hepatic insulin sensitivity are all consequences of the excess release of free fatty acids [57].

Clinical evidence strongly supports the critical role of adipose dysfunction in hepatic IR by establishing that adipose mass by calorie restriction, pharmacologic treatments, or lifestyle adjustment consistently improves systemic insulin sensitivity and lowers hepatic glucose output. MPs/NPs could exacerbate this immunometabolic imbalance, according to recent studies. MPs increase hepatic inflammatory signalling and may exacerbate systemic IR by disrupting the integrity of the GI-barrier, causing dysbiosis, and promoting macrophage-driven inflammation along the GI–liver axis [61,62]. Significantly, polymer type has the potential to influence metabolic toxicity: highly hydrophobic polymers such as polyethylene (PE) and polypropylene (PP) preferentially accumulate in adipose tissue, where they impede adipose insulin signalling by promoting TLR4–NF κB activation, macrophage infiltration, and adipocyte hypertrophy [61,62].

Conversely, polystyrene (PS), which possesses aromatic ring structures and a greater surface, causes hepatocytes to undergo more oxidative stress and mitochondrial dysfunction, which exacerbates hepatic IR and more strongly inhibits the IRS 1/AKT pathway [63].

While low-density polymers (such as PE and PP) exhibit longer retention in adipose depots, denser polymers (for example, PS and Polyethylene Terephthalate, PET) rapidly translocate to the liver, enhancing inflammatory signalling and steatosis [63]. The hypothesis that MPs function as polymer-specific “metabolic disruptors,” able to alter the adipose–liver axis and perhaps worsen IR in T2D, is supported by all of this evidence. The different toxicokinetic and toxicodynamic characteristics of these plastic particles across target metabolic tissues are methodically compiled in Table 1 to offer an organised summary of their unique multi-organ dynamics.

**Figure 3:**
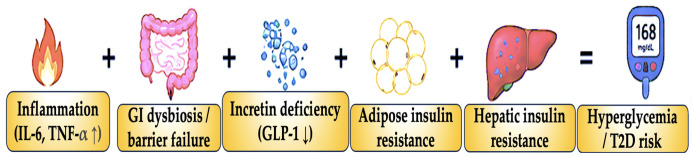
Convergence of pathways leading to T2D.

It is evident from analysing these tissue-specific dynamics that the metabolic hazard MP/NP exposure extends far beyond T2D as an independent endpoint (Figure 3).

Instead, the broader pathophysiological framework of metabolic syndrome (MetS) should incorporate environmental plastic particles and their chemical payloads [64,65]. MP/NP bioaccumulation may function as a shared upstream driver that simultaneously exacerbates visceral obesity, fuels insulin resistance, and accelerates the development of metabolic dysfunction-associated steatotic liver disease (MASLD/NAFLD) through the coordination of chronic systemic meta-inflammation, accelerated lipolysis, and endoplasmic reticulum stress [64,65,66].

## 11. Limitations of Current Evidence and Future Outlook

In order to preserve toxicological transparency, several types of substantial limitations must be recognised, even if the suggested mechanistic connections between MP/NP exposure and T2D pathogenesis are based on well-established physiological pathways. First, the existing paradigm is primarily hypothesis-driven and mainly depends on animal and in vitro models, which cannot accurately represent chronic metabolic reactions in humans. Second, pristine, spherical PS particles are used in several scientific studies at concentrations that frequently surpass reasonable human ambient exposure thresholds. Conversely, extremely heterogeneous, chemically damaged plastic shards consisting of several polymer types (PE, PP, and PET) and different additive mixtures are what humans are exposed to in real life. Third, significant, long-term human epidemiological cohorts demonstrating a direct, causal dose–response association between plastic body burden and T2D incidence are entirely absent, despite human biomonitoring evidence confirming the existence of MPs/NPs in circulating blood and tissues. Standardised interventional and prospective clinical designs must be accorded top priority in future studies in order to verify these experimental findings in human settings.

## 12. Conclusions

Emerging experimental models suggest that several biological pathways that are crucial to the early pathophysiology of type 2 diabetes may be modulated by MP/NP exposure, including hepatic inflammation, excess glucagon, intestinal barrier failure, microbiome remodelling, insufficient incretin signalling, and systemic insulin resistance. Early metabolic anomalies observed in exposed populations can be described coherently by the convergence of these pathways. A testable intervention technique with possible therapeutic significance is provided by the proposed concept of MP drainage. In order to evaluate compounds that could decrease intestinal MP burden and mitigate metabolic disturbance, translational research and clinical trials are required.

## Figures and Tables

**Table 1:** Differential toxicokinetic and toxicodynamic profiles of MPs/NPs across target metabolic tissues.

Polymer Type	Primary Cellular Target	Primary Molecular Mechanism	Resulting Metabolic Dysfunction	Supporting Level of Evidence
MPs	Intestinal epithelium, enteroendocrine L-cells	Mechanical abrasion, tight junction disruption, and altered luminal bile acid sensing	Reduced GLP-1 secretion, postprandial glycaemic excursions	In vivo (rodent models), in vitro (organoids)
NPs	Systemic circulation, hepatocytes, adipocytes	Passive lipid bilayer permeation, bio-corona formation, ROS generation	Intracellular oxidative stress, lysosomal membrane permeabilisation	In vitro (human cell lines), human biomonitoring (blood/placenta)
PE/PP	White Adipose Tissue (WAT)	Selective hydrophobic partitioning, TLR40NF-kB pathway activation	Adipocyte hypertrophy, enhanced lipolysis, MT macrophage polarisation	Preliminary in vivo animal biodistribution models
PS	Hepatic parenchyma (Kupffer cells)	High surface-to-volume ratio due to aromatic rings, mitochondrial damage	Inhibited IRS-1/Akt pathway, accelerated hepatic gluconeogenesis	Extensive in vitro models, acute murine exposure assays

## Data Availability

No new data were created or analysed in this study. Data sharing is not applicable to this article.

## Abbreviations

The following abbreviations are used in this manuscript:

Akt	Protein Kinase B
EDCs	Endocrine-Disrupting Chemicals
EVs	Extracellular Vesicles
GI	Gastrointestinal Tract
GLUT4	Glucose Transporter 4
HDL	High-Density Lipoproteins
IL-6	Interleukin-6
IR	Insulin Resistance
JNK	c-Jun N-terminal Kinase
LMP	Lysosomal Membrane Permeabilisation
LPS	Lipopolysaccharides
MASLD/NAFLD	Metabolic Dysfunction-Associated Steatotic Liver Disease
MetS	Metabolic Syndrome
MPs	Microplastics
NPs	Nanoplastics
PAMPs	Pathogen-Associated Molecular Patterns
PBPK	Physiological-Based Pharmacokinetics
PCC	Procambarus Clarkii Chitosan
PE	Polyethylene
PET	Polyethylene Terephthalate
PI3K	Phosphoinositide 3-Kinase (PI3K)
POPs	Persistent Organic Pollutants
PP	Polypropylene
PS	Polystyrene
PS-NPs	Polystyrene Nanoplastics
RCTs	Randomised Controlled Trials
ROS	Reactive Oxygen Species
SCFAs	Short Chain Fatty Acids
TLR4	Toll-like Receptor 4
TNF-α	Tumour Necrosis Factor-Alpha
T2D	Type 2 Diabetes
WAT	White Adipose Tissue
